# Emotion recognition, alexithymia, empathy, and emotion regulation in women with anorexia nervosa

**DOI:** 10.1007/s40519-022-01496-2

**Published:** 2022-10-18

**Authors:** Emma Saure, Anu Raevuori, Marja Laasonen, Tuulia Lepistö-Paisley

**Affiliations:** 1 Department of Psychology and Logopedics, Faculty of Medicine, University of Helsinki, P.O. Box 21, 00014 Helsinki, Finland; 2 BABA Center and Department of Clinical Neurophysiology, Children’s Hospital, Helsinki University Hospital and University of Helsinki, Helsinki, Finland; 3 Department of Public Health, University of Helsinki, Helsinki, Finland; 4 Department of Adolescent Psychiatry, Helsinki University Hospital, Helsinki, Finland; 5 School of Humanities, Philosophical Faculty, University of Eastern Finland, Joensuu, Finland; 6 Department of Child Neurology, Helsinki University Hospital and University of Helsinki, Helsinki, Finland

**Keywords:** Anorexia nervosa, Feeding and eating disorders, Emotion recognition, Alexithymia, Empathy, Emotion regulation

## Abstract

**Purpose:**

Anorexia nervosa (AN) is associated with challenges in recognizing, understanding, and interpreting one’s own and other’s emotional states, feelings, and thoughts. It is unknown whether difficulties in emotion processing occur independently of common comorbid symptoms of AN and predict acute eating disorder characteristics. We aimed to examine emotion recognition, alexithymia, emotion regulation, and empathy in individuals with AN and to assess whether these predict eating disorder symptoms independently from comorbid symptoms.

**Methods:**

Participants included 42 women with AN and 40 healthy control (HC) women between 18–30 years. Basic and complex emotion recognition was assessed with face photos and video clips. Alexithymia, empathy, emotion regulation, and comorbid symptoms (anxiety, depressive, and obsessive–compulsive symptoms and ASD traits) were assessed with self-assessment questionnaires.

**Results:**

Participants with AN exhibited difficulties in basic and complex emotion recognition, as well as increased alexithymia, decreased empathy, and challenges in emotion regulation when compared to HCs. After controlling for comorbid symptoms, differences remained only in complex emotion recognition. Challenges in emotion recognition were associated with lower body mass index, and increased alexithymia was associated with increased eating disorder symptoms. Increased challenges in emotion regulation were associated with a shorter duration of illness, higher body mass index, and increased eating disorder symptoms.

**Conclusions:**

Participants with AN displayed widespread deficit in emotion processing, but only challenges in complex emotion recognition occurred independently from comorbid symptoms. Deficits in emotion processing may contribute to the illness severity and thus could be an important treatment target.

**Level of Evidence:**

Level III, case-control analytic study.

**Supplementary Information:**

The online version contains supplementary material available at 10.1007/s40519-022-01496-2.

## Introduction

Anorexia nervosa (AN) is associated with challenges in recognizing, understanding, and interpreting one’s own and other’s emotional states, feelings, and thoughts. The ability to recognize and interpret other’s mental states is critical for organizing and directing social cognition and behavior, as well as for adapting flexibly to different social situations [1, 2]. Poor ability to recognize and interpret others’ mental states may thus predispose to difficulties in interpersonal relationships, which are an important risk factor for AN [1, 3]. Furthermore, the ability to identify and process one’s own mental states is critical for successful emotion identification and regulation, and difficulties in these are associated with maladaptive behavior that is common in individuals with AN [4, 5]. In this study, we aimed to examine different aspects of emotion processing in individuals with AN, that is, emotion and mental states recognition, alexithymia, empathy, and emotion regulation.

Emotion recognition and recognizing others’ mental states are reported to be challenging for individuals with AN [e.g., 2, 6]. Challenges in emotion recognition in childhood are associated with later AN, thus suggesting that poor capability in emotion recognition may predispose to AN [3]. Some studies have suggested that individuals with AN may have an intact ability to recognize basic emotions (anger, fear, sadness, happiness, surprise, disgust). Challenges may emerge only in the recognition of complex emotions and mental states (e.g., ashamed, bored, disappointed, excited, frustrated, and jealous) as this process is more demanding and requires a more advanced capability to understand and perceive other’s emotional expressions compared to the recognition of basic emotions [7, 8]. It is proposed that individuals with AN can recognize valence (i.e., whether the emotion is positive or negative) and this, in turn, helps them to use one’s conceptual knowledge for identifying emotions [9]. This strategy may be effective when identifying a limited repertoire of basic emotions, but in the context of complex emotions and mental states, it would be impossible to learn all the different concepts in which these may occur. Therefore, we aimed to examine whether individuals with AN have challenges in complex emotion recognition using photos with a whole face and video clips. These correspond better to real-life situations than photos containing only the eye area, which have been used in previous studies [2].

Alexithymia is reported to be common in individuals with AN [e.g., 2]. Alexithymia refers to the inability to identify and describe one’s own emotions, and it is suggested to relate to general deficits in interpreting interoceptive signals, such as hunger, tiredness, or arousal [10, 11]. Alexithymia is associated with eating disorder symptoms among individuals with short-duration AN [4]. Further, alexithymia is associated with social anxiety and avoidance, both possible risk factors for the prolongation of AN [4]. However, it is unknown if alexithymia contributes to the severity of AN symptoms among adults or whether it is associated with AN prolongation.

Empathy refers to the ability to understand how someone else is feeling and to relate to others´ thoughts and mentals states [12]. Empathy is essential for responding appropriately to other’s emotional states, and thus, it is also a critical ability for successful social interaction. Individuals with AN are suggested to exhibit low empathy [13]. It is known that struggles in interpersonal relationships are an important risk factor for AN, but the relationship between empathy abilities and the severity of AN has not been investigated.

Emotion regulation is challenging for individuals with AN and is suggested to contribute to AN symptoms [e.g., 14]. Emotion regulation refers to the ability to modify, regulate and monitor one’s own emotional reactions and behavior. Individuals with AN use more maladaptive emotion regulation strategies, and it is suggested that they use eating disorder symptoms as a way to regulate their emotions when adaptive strategies are lacking [5, 14]. Further, it has also been proposed that neurotransmitter changes caused by starvation may promote emotion regulation capacity in individuals with AN [5]. It is thus possible that emotion regulation challenges contribute to acute eating disorder symptoms and the severity of AN.

The presence of comorbid psychiatric and neuropsychiatric symptoms, common in individuals with AN, affects emotion recognition, alexithymia, empathy, and emotion regulation [15–18]. For example, increased emotion recognition challenges in individuals with AN are associated with higher obsessive–compulsive symptoms [15]. Furthermore, it has been reported that increased depression and anxiety symptoms are associated with higher alexithymia and may mediate the occurrence of alexithymia in individuals with AN [11, 17]. In addition, difficulties in emotion recognition, alexithymia, empathy, and emotion regulation are also characteristics of autism spectrum disorder (ASD). Elevated ASD traits and diagnosed ASD are common in individuals with AN, and challenges in emotion recognition and low empathy are associated with elevated ASD traits in individuals with AN [18, 19]. Previous studies investigating emotion recognition, alexithymia, empathy, and emotion regulation have reported heterogeneous results among participants with AN, and one important reason for this may be the variability of confounding variables as discussed above [14]. This can also be one reason for difficulties in research replication in these emotion processing dimensions in individuals with AN. Therefore, it is crucial to control these comorbid symptoms when assessing basic and complex emotion recognition, alexithymia, empathy, and emotion regulation in individuals with AN. To our best knowledge, there are no previous studies that would have examined how the combined effect of comorbid variables affects emotion processing abilities in individuals with AN.

To conclude, individuals with AN appear to have deficits in basic and complex emotion recognition, alexithymia, empathy, and emotion regulation, but it is not clear whether these deficits reflect the combined effect of comorbid symptoms in individuals with AN. Further, challenges in emotion processing may predict the severity of AN. This study aimed to examine 1) basic and complex emotion recognition, alexithymia, empathy, and emotion regulation in individuals with AN as compared to HCs when controlling comorbid symptoms (i.e., anxiety, depressive, and obsessive–compulsive symptoms and ASD traits), and 2) whether possible challenges in basic and complex emotion recognition, alexithymia, empathy, and emotion regulation are associated with the severity of AN, as measured with BMI, duration of illness, and number of eating disorder symptoms.

## Methods

### Participants

Participants with AN were recruited via The Eating Disorder Association of Finland and Turku University Hospital. All individuals with AN had been diagnosed by professionals and fulfilled the ICD-10 diagnostic criteria of AN or atypical AN (F50.0 and F50.1), in which AN corresponds to the DSM-5 definition of AN (307.1), and atypical AN corresponds to the DSM-5 definition of other specified feeding and eating disorder (307.59) [20, 21]. The healthy controls were recruited via the University of Helsinki e-mailing lists. Exclusion criteria for all the participants were history of psychosis, head trauma with unconsciousness, substance abuse, neuropsychiatric or neurological disorder, learning disability, or Full-Scale Intelligence Quotient (FSIQ), Verbal Comprehension Index (VCI), or Perceptual Reasoning Index (PRI) under 70. Furthermore, the HCs were required to have no history of mental disorders. Approval for the study was obtained from the Ethics committee of Helsinki Uusimaa Hospital District (HUS/1886/2017). The participants participated voluntarily after providing informed written consent for the study. The final sample comprised 82 participants: 40 HCs and 42 individuals with AN (AN *n* = 28, atypical AN *n* = 14). Three individuals were excluded from the original HC group (*n* = 43): one had a PRI under 70, one reported having a diagnosed mental health problem, and one reported having a diagnosed neurological condition. The age range of the participants was 18–30 years. Among the AN group, 15 participants reported having psychopharmacological medication, and 21 participants reported having one or more comorbid psychiatric disorders. The most common comorbid conditions were anxiety disorders and major depression. The characteristics of participants in both groups (AN, HC) are presented in Table 1. See also Supplementary Table 1 for descriptive statistics of background variables.

**Table 1:** Means and standard deviations and test statistics of clinical and comorbid characteristics of AN and HC groups

	AN (*n* = 42)		HC (*n* = 40)		Test statistic	*p* value
	Mean	(S.D.)	Mean	(S.D.)		
Age (years)	23.61	(3.69)	23.10	(3.03)	*U* = 877.500	NS
Education (years)	14.21	(2.43)	14.15	(1.82)	*U* = 819.000	NS
FSIQ	108.31	(17.15)	109.68	(13.00)	*t* (80) = 0.405	NS
PRI	105.33	(15.91)	106.10	(15.02)	*t* (80) = 0.224	NS
VCI	108.36	(14.37)	110.48	(13.91)	*t* (80) = 0.677	NS
BMI	17.00	(2.64)	21.61	(1.22)	*t* (78) = 10.013	< 0.001
Duration of illness	7.46	(3.65)	N/A	N/A	N/A	N/A
Eating* D*isorder symptoms total (EDE-Q)	85.86	(33.08)	9.32	(7.11)	*U* = 1679.000	< 0.001
Anxiety symptoms (BAI)	19.31	(10.22)	4.83	(3.03)	*t* (80) =8.786	< 0.001
Depressive symptoms (BDI-II)	23.52	(12.96)	2.45	(3.19)	*U* = 1611.500	< 0.001
Obsessive–compulsive symptoms (OCI-R)	22.93	(11.17)	7.43	(5.41)	*U* = 1549.000	< 0.001
Autism spectrum traits (AQ)	21.57	(9.29)	10.90	(6.32)	*U* = 1392.500	< 0.001

*FSIQ* full-scale intelligence quotient, *VCI* verbal comprehension index, *PRI* perceptual reasoning index, *BMI* body mass index, *EDE-Q* eating disorder examination questionnaire, *BAI* beck anxiety inventory, *BDI-II* beck depressive inventory-II, *OCI-R* obsessive–compulsive inventory-revised, *AQ* autism quotient, *N/A* not applicable, *NS* not significant

## Measures

### Background information

The participants filled in a self-report background questionnaire. They were asked about their date of birth, education in years, psychiatric and neurologic conditions, psychopharmacological medication, weight, and height. BMI was calculated using self-reported weight and height.

### Wechsler Abbreviated Scale of Intelligence -IV (WASI -IV)

The participants’ cognitive ability was assessed using WASI-IV consisting of the following subtests: vocabulary, similarities, block design, and matrix reasoning. It produces verbal, performance, and full-scale intelligence quotient scores (VCI, PRI, FSIQ) [22]. These quotients were formed based on Wechsler Adult Intelligence Scale-IV (WAIS-IV) [23], as there is no official edition of the WASI in Finland.

### Basic and complex emotion recognition

EU-emotion stimulus set was used to investigate both basic and complex emotion recognition from facial expressions [24, 25]. The set consists of photos of actors expressing six basic emotions (afraid, sad, angry, disgusted, happy, surprised) and 15 complex emotions / mental states (ashamed, bored, disappointed, excited, frustrated, hurt, interested, jealous, joking, kind, proud, sneaky, unfriendly, worried, neutral), totaling 126 face photos. In the photos, the actor’s upper body is shown on a white background. The photo set included 41 photos of children (girls *n* = 10, boys *n* = 31; Caucasian *n* = 25, non-Caucasian *n* = 16), 29 photos of teenagers (girls n = 21, boys = 8, all Caucasian), 40 photos of adults (women *n* = 29, men *n* = 11; Caucasian *n* = 10, non-Caucasian *n* = 30) and 16 photos of elderly (women *n* = 7, men *n* = 9, all Caucasian). The photos were shown on a computer screen in a randomized order, which was different for each participant, along with six response options. Participants had to select the response they thought described the facial expression best. The six response options consisted of the target emotion/mental state, four control emotions/mental states, and “none of the above” response option. Photos were presented on the screen until the participants selected one response option. The response was rated as 1 when it was correct and 0 when it was incorrect or “none of the above”. A higher score indicates better performance. The maximum score for basic emotions is 36 (six different emotions, including six photos of each emotion), and for complex emotions 90 (15 different emotions, including six photos of each emotion). Furthermore, the Cambridge Mindreading (CAM) battery was used to investigate complex emotion recognition from short video clips [7]. The battery includes 3–5-s-long silent video clips of adult actors expressing complex emotions/mental states (resentful, stern, grave, subdued, exonerated, uneasy, empathic, vibrant, admiring, lured, subservient, appalled, confronted, intimate, insincere, restless, appealing, mortified, guarded, distaste, nostalgic, reassured), totaling 52 video clips (2–3 videos of each emotion). The actor’s upper body is shown on a white background. The video set included 42 videos of adults (women n = 25, men *n* = 17; Caucasian *n* = 23, non-Caucasian *n* = 19) and 10 videos of elderly (women *n* = 5, men *n* = 5, all Caucasian). The video clips were presented on a computer screen in a randomized order which was different for each participant. After each clip, there were four response choices, including the target emotion and three other emotions, from which the participants had to select one. The response options were presented on the screen until the participants had responded. The response was rated as 1 when it was correct and 0 when it was incorrect. A higher score indicates better performance, and the maximum score is 52. We decided to include both still photos and videoclips since we wanted to find out if there are differences between basic and complex emotion recognition, and there were no suitable video clips showing basic emotions available. However, as videos reflect real-life situations better than photos, we wanted to include the video clips as well. The Cronbach’s Alpha of emotion recognition tasks in this study was *α* = 0.627.

### Toronto Alexithymia Scale (TAS-20)

TAS-20 is a standardized and widely used scale for measuring alexithymia in the areas of difficulties in identifying feelings, difficulties in describing feelings to others, and externally oriented thinking [26]. It contains 20 self-report items. A higher score indicates higher alexithymia. The maximum score is 100. The Cronbach’s Alpha of TAS-20 was *α* = 0.766.

### Empathy Quotient (EQ)

EQ is a questionnaire that measures empathizing. It consists of 40 self-report questions and measures affective and cognitive empathy [12]. A higher score indicates better empathizing. The maximum score is 80. The Cronbach’s Alpha of EQ was *α* = 0.902.

### Difficulty in Emotion Regulation Scale (DERS)

DERS is a validated scale aimed to measure emotion regulation in the areas of non-acceptance of emotions, the difficulty of engaging in goal-directed behavior while experiencing negative emotions, impulse control difficulties, lack of emotional awareness, limited access to regulate emotions, and lack of emotional clarity [27]. DERS contains 36 self-report items. A higher score indicates more emotion regulation difficulties. The maximum score is 180. The Cronbach’s Alpha of DERS was *α* = 0.870.

### Eating Disorder Examination Questionnaire (EDE-Q)

Eating Disorder Symptoms were assessed with EDE-Q [28], which is a self-report questionnaire consisting of 36 questions. It measures restraint, eating concerns, shape concerns, and weight concerns. A higher score indicates more eating disorder symptoms. The Cronbach’s Alpha of EDE-Q was *α* = 0.943.

### Beck Anxiety Inventory (BAI)

BAI was used to measure anxiety symptoms. BAI is a widely used self-report questionnaire that consists of 21 questions measuring anxiety symptoms [29]. A higher score indicates more anxiety symptoms. The Cronbach’s Alpha of BAI was *α* = 0.916.

### Beck Depressive Inventory-II (BDI-II)

BDI-II was used to evaluate depressive symptoms [30]. BDI-II is a widely used self-report questionnaire containing 21 questions. A higher score indicates more depressive symptoms. The Cronbach’s Alpha of BDI-II was *α* = 0.961.

### Obsessive–Compulsive Inventory-Revised (OCI-R)

OCI-R was used to measure obsessive–compulsive symptoms [31]. OCI-R is a widely used self-report questionnaire containing 18 questions measuring washing, checking, ordering, obsessing, hoarding, and mental neutralizing. A higher score indicates more obsessive–compulsive symptoms. The Cronbach’s Alpha of OCI-R was *α* = 0.731.

### Autism Quotient (AQ)

ASD traits were measured using AQ, which is a widely used scale containing 50 self-report items. It measures ASD traits in the areas of social skills, attention switching, attention to details, communication, and imagination. A higher score indicates increased ASD traits. The Cronbach’s Alpha of AQ was *α* = 0.873.

### Data analysis

Power analysis revealed that the sample size was sufficient for emotion recognition, alexithymia, and emotion regulation (80% power with alpha set at *p* < 0.05) [32]. In the context of empathy, our study was slightly unpowered as about 55 participants would have been required in both groups. The Statistical Package for the Social Sciences, version 26.0 [33], was used to analyze the data. The normality of variables in the two samples was assessed with histograms and Shapiro–Wilk tests. Homogeneity of variance was assessed with Levene’s test. Linearity of dependent variables (emotion recognition, alexithymia, empathy, and emotion regulation) and covariates (AQ, BAI, BDI-II, OCI-R) were assessed with scatter plots and test for linearity.

Independent samples *t* test was used to calculate group differences for background variables that were distributed normally in both groups (FSIQ, VCI, PRI, BMI, BAI), and Mann–Whitney *U* test was used to calculate group differences for other background variables (education, age, EDE-Q, OCI-R, BD-II, AQ). Regarding the variables that were the targets of this study, multivariate analysis of variance (MANOVA) and analysis of variance (ANOVA) as well as multivariate analysis of covariance (MANCOVA) and analysis of covariance (ANCOVA) were used for variables that fulfilled the criteria of these tests (emotion recognition tasks and EQ). Kruskal–Wallis test and non-parametric analysis of covariance (QUADE) were used for variables that did not fulfill the criteria of parametric tests (alexithymia and emotion regulation). The results of the emotion recognition tasks and the scores of TAS-20, EQ, and DERS were included in analyses as dependent variables and group (AN and HC) as the independent variable. In the ANCOVA and QUADE, comorbid symptoms measured by BAI, BDI-II, OCI-R, and AQ were included as covariates. In analyses, effect size (partial *η*^2^) 0.01 indicates a small effect, 0.06 indicates a medium effect, and 0.14 or higher indicates a large effect. In the emotion recognition from face photos, the data of four HC participants and six AN participants, and in the emotion recognition from video clips, the data of two HC participants and six AN participants were missing because of technical problems. The BMI data were missing from two AN participants. No other data were missing. Missing information was not replaced, and thus, participants whose data were missing were not included in the analyses including these missing variables.

Linear regression analyses were used to identify whether the group (AN or HC) and emotion recognition, alexithymia, empathy, and emotion regulation predict the BMI, duration of illness, and scores of EDE-Q. Before the analyses, the scores of the emotion recognition tasks (basic and complex emotion recognition from face photos and complex emotion recognition from videos) were summed up and combined under one variable representing overall emotion recognition. The internal consistency of the emotion recognition composite was acceptable when investigated with an inter-item correlation matrix and Cronbach’s Alpha (0.627). The group was included at the first step since there was a significant group difference in the variables included in the analyses, and we wanted to find out if difficulties in emotion recognition, alexithymia, empathy, and emotion regulation predict the severity of AN after the effect of the group is considered. Emotion recognition, alexithymia, empathy, and emotion regulation were analyzed in separate regression analyses.

## Results

### Emotion recognition

In the first analysis, we examined with MANOVA whether there were differences between the AN and HC groups in emotion recognition. The AN group performed significantly worse than the HC group in emotion recognition [*F* (3,68) = 11.026, *p* < 0.001, Wilks’ *λ* = 0.673, partial *η*^2^ = 0.327]. Follow-up ANOVAs revealed significant group differences in all emotion recognition tasks: as compared to HCs, the participants with AN exhibited poorer basic emotion recognition from face photos (*p* = 0.007, partial *η*^2^ = 0.098), poorer complex emotion recognition from face photos (*p* = 0.002, partial *η*^2^ = 0.123), and poorer complex emotion recognition from video clips (*p* < 0.001, partial *η*^2^ = 0.294; see Table 2 and for the descriptive statistic, see Supplementary Table 2). After controlling covariates in MANCOVA, it was found that the AN group performed still significantly worse than the HC group in emotion recognition [*F* (3,64) = 3.333, *p* = 0.025, Wilks’ *λ* = 0.865, partial *η*^2^ = 0.135]. Follow-up ANOVAs revealed that the significant group difference disappeared in basic emotion recognition (*p* = 0.150), but participants with AN still exhibited significantly poorer complex emotion recognition than HCs from face photos (*p* = 0.043, partial *η*^2^ = 0.060), and from video clips (*p* = 0.005, partial *η*^2^ = 0.112).

**Table 2:** Means, standard deviations and test statistics of emotion recognition tasks and the TAS-20, EQ, and DERS questionnaires

	AN (*n* = 42)		HC (*n* = 40)		ANOVA or Kruskal–Wallis (comparison without covariates)		ANOCOVA or QUADE (comparison with covariates)	
	Mean	(S.D.)	Mean	(S.D.)	Test statistic	*p* value	Test statistic	*p* value
Basic emotion recognition from face pictures	25.50	(4.29)	27.97	(3.24)	*F* (1, 70) = 7.625	0.007	*F* (1, 66) = 2.124	NS
Complex emotion recognition from face pictures	47.22	7.43	52.28	(6.18)	*F* (1, 70) = 9.856	0.002	*F* (1, 66) = 4.238	0.043
Complex emotion recognition from face videos	32.69	(4.41)	37.63	(3.76)	*F* (1, 70) = 29.097	< 0.001	*F* (1, 66) = 8.311	0.005
TAS-20 (toronto alexithymia scale)	55.93	(11.99)	36.95	(7.54)	*H *(1) = 40.793	< 0.001	*F* (1, 80) = 1.035	NS
EQ (empathy quotient)	48.88	(12.99)	54.93	(11.21)	*F* (1, 80) = 5.064	0.027	*F* (1,76) = 0.368	NS
DERS (difficulties in emotion regulation scale)	106.95	(22.95)	68.50	(14.71)	*H* (1) = 46.762	< 0.001	*F* (1, 80) = 0.534	NS

*NS* not significant

### Alexithymia

Kruskal–Wallis test revealed that the participants with AN exhibited significantly higher alexithymia than the HCs (*p* < 0.001, see Table 2). After controlling for the covariates in a QUADE analysis, the significant difference between the groups disappeared (*p* = 0.312).

### Empathy

The participants with AN exhibited significantly lower empathizing than HCs in the ANOVA (*p* = 0.027, partial *η*^2^ = 0.060, see Table 2). After controlling for covariates in ANCOVA analyses, the significant difference between the groups disappeared (*p* = 0.546).

### Emotion regulation

Kruskal–Wallis test showed that the AN participants exhibited significantly more impairment in emotion regulation than the HCs (*p* < 0.001, see Table 2). After controlling for the covariates in QUADE analysis, the significant difference between the groups disappeared (*p* = 0.467).

### Difficulties in emotion recognition, alexithymia, empathy, and emotion regulation as predictors of characteristics of anorexia nervosa

Linear regression revealed that the group at step 1 was a significant predictor for BMI (*p* < 0.001). Emotion recognition difficulties at step 2 significantly increased the accuracy of prediction (*p* < 0.001; p change = 0.008). In addition, emotion regulation difficulties at step 2 significantly increased the accuracy of prediction (*p* < 0.001, *p* change = 0.044, See Table 3). For the duration of illness, the group was a significant predictor at step 1 (*p* < 0.001). Only emotion regulation significantly increased the accuracy of prediction at step 2 (*p* < 0.001, *p* change = 0.017). For the eating disorder symptoms, the group was a significant predictor at step 1 (*p* < 0.001). Alexithymia significantly increased the accuracy of prediction at step 2 (*p* < 0.001, *p* change = 0.011). Moreover, emotion regulation difficulties significantly increased the accuracy of prediction at step 2 (*p* < 0.001, *p* change < 0.001).

**Table 3:** Coefficient of determination (R2) for each step and changes in R2 are presented in table for emotion processing dimensions (emotion recognition, alexithymia, empathy, emotion regulation)

	BMI	Duration of illness	EDE-Q
Emotion recognition			
First step: only group, significance of model	*F* (1,69) = 88.683, *R*^2^ = 0.562, *p* < 0.001	*F* (1,70) = 146.056, *R*^2^ = 0.676, *p* < 0.001	*F* (1,70) = 179.389, *R*^2^ = 0.719, *p* < 0.001
Second step: group and emotion recognition, significance of *R*^2^ change	*F* change (1,68) = 7.452, *R*^2^ change = 0.043, *p* change = 0.008	*F* change (1,69) = 1.364, *R*^2^ change = 0.006, *p* change = NS	*F* change (1,69) = 0.081, *R*^2^ change = 0.000, *p* change = NS
Second step: group and emotion recognition, significance of model	*F* (2,68) = 52.214, *R*^2^ = 0.606, *p* < 0.001	*F* (2,69) = 74.089 *R*^2^ = 0.682, *p* < 0.001	*F* (2,69) = 88.557, *R*^2^ = 0.720, *p* < 0.001
Alexithymia			
First step: only group, significance of model	*F* (1,78) = 100.250, *R*^2^ = 0.562, *p* < 0.001	*F* (1,80) = 166.921, *R*^2^ = 0.676, *p* < 0.001	*F* (1,80) = 205.016, *R*^2^ = 0.719, *p* < 0.001
Second step: group and alexithymia, significance of *R*^2^ change	*F* change (1,77) = 0.100 *R*^2^ change = 0.001, *p* change = NS	*F* change (1,79) = 0.006 *R*^2^ change = 0.000, *p* change = NS	*F* change (1,79) = 6.760, *R*^2^ = 0.022, *p* change = 0.011
Second step: group and alexithymia, significance of model	*F* (2,77) = 49.597, *R*^2^ = 0.563, *p* < 0.001	*F* (12,79) = 82.427, *R*^2^ = 0.676, *p* < 0.001	*F F* 2,79) = 113.269, *R*^2^ = 0.735, *p* < 0.001
Empathy			
First step: only group, significance of model	*F* (1,78) = 100.250, *R*^2^ = 0.562, *p* < 0.001	*F* (1,80) = 166.921, *R*^2^ = 0.676, *p* < 0.001	*F* (1,80) = 205.016, *R*^2^ = 0.719, *p* < 0.001
Second step: group and empathy, significance of *R*^2^ change	*F* change (1,77) = 1.571, *R*^2^ change = 0.009, *p* change = NS	*F* change (1,77) = 0.079, *R*^2^ change = 0.000, *p* change = NS	*F* change (1,80) = 3.201, *R*^2^ change = 0.011, *p* change = NS
	*F* = (2,77) = 51.277, *R*^2^ = 0.571, *p* < 0.001	*F* (2,79) = 82.539, *R*^2^ = 0.676, *p* = < 0.001	*F* (2,79) = 106.929, *R*^2^ = 0.730, *p* < 0.001
Second step: group and empathy, significance of model			
Emotion regulation			
First step: only group, significance of model	*F* (1,78) = 100.250, *R*^2^ = 0.562, *p* < 0.001	*F* (1,80) = 166.921, *R*^2^ = 0.676 *p* < 0.001	*F* (1,80) = 205.016, *R*^2^ = 0.719, *p* < 0.001
Second step: group and emotion regulation, significance of *R*^2^ change	*F* change (1,77) = 4.183, *R*^2^ change = 0.023, p change = 0.044	*F* change (1,79) = 5.924, *R*^2^ change = 0.023, *p* change = 0.017	*F* change (1,79) = 23.962, *R*^2^ change = 0.065, *p* change < 0.001
Second step: group and emotion regulation, significance of model	*F* (2,77) = 54.263, *R*^2^ = 0.585, *p* < 0.001	*F* (2,79) = 91.559, *R*^2^ = 0.699, *p* < 0.001	*F* (2,79) = 143.911, *R*^2^ = 0.785, *p* < 0.001

*BMI* body mass index, *EDE-Q* eating disorder examination questionnaire

## Discussion

This study investigated emotion recognition, alexithymia, empathy, and emotion regulation in individuals with AN compared to HCs. Women with AN performed poorer in basic and complex emotion recognition tasks and exhibited increased alexithymia, less empathy, and more emotion regulation difficulties when compared to HCs. However, in this study, the group differences between women with AN and HCs disappeared in most of the investigated dimensions when controlling for comorbid symptoms, that is, anxiety, depressive, and obsessive–compulsive disorder symptoms as well as ASD traits. Our findings suggest that difficulties in basic emotion recognition, high alexithymia, low empathy, and challenges in emotion regulation in individuals with AN may reflect confounding symptoms and may be associated with a broader psychopathology network that is shared with different mental or neurodevelopmental disorders [5, 34]. Our findings also strengthen previous findings that anxiety, depression, obsessive–compulsive symptoms, and high ASD traits mediate the occurrence of difficulties in basic and complex emotion recognition, alexithymia, empathy, and emotion regulation in individuals with AN [14, 15, 17–19]. Therefore, inconsistent results of previous studies and lack of replication may reflect varying comorbid characteristics among participants with AN that have not usually been controlled.

Interestingly, we found that individuals with AN have challenges in complex emotion and mental state recognition when compared to HCs and that these challenges occur independently of anxiety, depressive, obsessive–compulsive symptoms, and ASD traits. Our findings are in line with previous evidence related to challenges in complex emotion recognition in individuals with AN [8]. These challenges in complex emotion recognition appear to be an endo-phenotype of AN since they have been found to exist also among the unaffected twins of individuals with AN [35]. In contrast, challenges in basic emotion recognition were not independent of the comorbid variables. A possible explanation for this is that by adulthood, individuals with AN have learned basic emotion recognition by developing compensatory strategies. Interestingly, in the context of ASD, it has been shown that adults with ASD have difficulties in recognizing complex emotions, whereas their ability to recognize basic emotions is intact [7]. The reason for this is suggested to be that the recognition of complex emotions requires higher cognitive processing and is thus more demanding and also more challenging learn [7]. The same may be true among individuals with AN, and our findings support the previous suggestions that individuals with AN may have difficulties specifically in recognizing and understanding complex emotions that require more advanced social cognition [8].

**Fig. 1:**
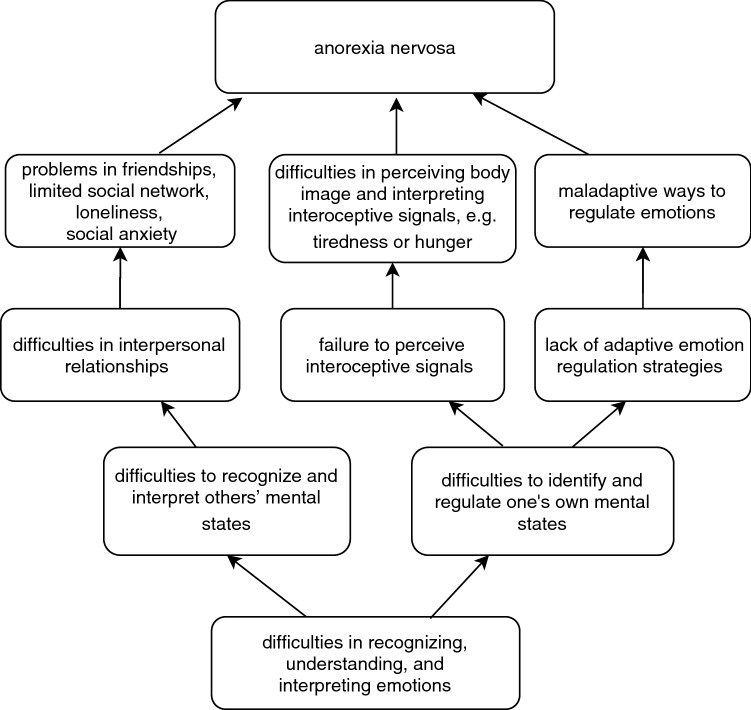
Possible ways how difficulties in emotion recognition, alexithymia, empathy, and emotion regulation may affect the development of AN

Another aim of the present study was to find out whether difficulties in emotion recognition, alexithymia, empathy, and emotion regulation are associated with the severity of AN. We found that emotion recognition difficulties predicted lower BMI, higher alexithymia predicted increased eating disorder symptoms, and increased emotion regulation difficulties predicted increased eating disorder symptoms and higher BMI. Our findings are in line with previous findings suggesting that difficulties in emotion recognition, alexithymia, and emotion regulation may contribute to maintaining and prolonging AN [1, 5, 6]. Emotion recognition difficulties may predispose one to adverse experiences in social situations, such as loneliness, bullying, and feeling different, which are all risk factors for AN and may contribute to the severity of AN [36]. Difficulties in emotion recognition are also related to mentalization ability, that is to the ability to understand both one’s own and the others behavior and think about how this behavior is related to underlying mental states [37]. Challenges in mentalization may lead to misinterpretations of social situations and thus predispose to struggles in interpersonal relationships. Further, alexithymia may reflect a general failure to perceive interoceptive and physiological information from the body that may translate into poorer recognition of hunger and satiety signals, as well as into difficulties in perceiving one’s body image, which could contribute to eating disorder symptoms [10]. Concerning emotion regulation, it has been suggested that AN is characterized by challenges in accepting aversive emotions and that eating disorder symptoms serve as a maladaptive strategy for regulating these emotions [5, 38]. Individuals with AN are found to eat less in response to negative emotions, and it is proposed that weight loss and dieting among individuals with AN may contribute to an improved sense of emotional control and therefore eating disorder symptoms help individuals with AN to regulate their emotions [5, 38]. Our finding that emotion regulation difficulties predicted higher eating disorder symptoms strengthens the previous evidence about the role of eating disorder symptoms as a way of regulating emotions. Our finding that lower BMI was associated with better emotion regulation abilities is also in line with previous studies suggesting that weight loss may strengthen feelings of control of one’s own emotions among individuals with acute AN [5]. See Fig. 1 for possible ways how challenges in recognizing, understanding, and interpreting emotions may affect the development of AN symptoms.

Surprisingly, we found that a shorter duration of illness was associated with greater difficulties in emotion regulation. There are some possible explanations for this. First, treatment is suggested to decrease emotion regulation difficulties [39], and those with prolonged AN have probably also had longer treatment periods during which they may have learned emotion regulation strategies. However, one study found that emotion regulation ability did not improve after treatment and weight restoration in individuals with AN [40], but in that study, the duration of AN or the duration of possible previous treatment periods were not reported, leaving the possibility that longer treatment or multiple treatment periods may improve emotion regulation. The mean duration of AN in our sample was over seven years, and therefore, it is likely that they had undergone long-lasting treatment or several treatment periods. Another possible explanation is that those who have greater emotion regulation difficulties develop some other eating disorder than AN in long-term follow-up. There is evidence of greater emotion regulation problems in individuals with bulimia nervosa (BN) or binge eating disorder (BED) than in individuals with AN [39]. Diagnostic crossover between AN and BN is suggested to happen at relatively early stages in the disease course of AN, and therefore, these individuals are not included among those with prolonged AN [41]. To conclude, difficulties in emotion recognition and high alexithymia may reinforce eating disorder behavior and increase the likelihood of poor outcomes. In the context of emotion regulation challenges, the relationship to eating disorder behavior seems to be complex and more research that also considers the effects of treatment and subtypes of eating disorders is needed.

### Strength and limitations

The main strengths of this study are that we controlled various comorbid symptoms and included both basic and complex emotion recognition tasks, as well as used not only photos but also videoclips in the emotion recognition task. The main limitation of the present study is that although basic and complex emotion recognition were assessed with objective emotion recognition tasks, alexithymia, empathy, and emotion regulation were measured with self-report questionnaires, where accurate reporting is dependent on participants’ capability to recognize, self-reflect, and verbalize these concepts. However, it has been shown that self-reported alexithymia correlates with objective psychophysiological measures of emotional arousal, suggesting that at least the self-report questionnaire measuring for alexithymia has good validity [42]. In the context of alexithymia and emotion regulation, we did not explore the subscales due to the small sample size and because there were no group differences in the total scores of these scales after comorbid variables were controlled. Future studies with bigger samples are needed to analyze whether there are group differences in the subscales of these instruments after controlling comorbid variables. Another limitation is that weight and height were self-reported, and some reporting bias may have occurred. Additionally, psychopharmacological medication or comorbid conditions were not controlled for analyses since these were very heterogeneous. Finally, the study included only females, and it is known that at least in the empathy questionnaire, females score higher than males in the general population [12]. Therefore, the results may not be generalizable to males with AN.

## Conclusion

Our study provides several valuable findings and adds up to the growing evidence of comprehensive difficulties in emotion processing among individuals with AN. First, we found out that individuals with AN have challenges in complex emotion recognition and that these challenges occur independently of comorbid symptoms. Second, we showed that individuals with AN have challenges in basic emotion recognition and increased alexithymia, lower empathy, and increased challenges in emotion regulation, but that these may reflect confounding symptoms. Lastly, we found that deficits in emotion recognition, alexithymia, and emotion regulation contribute to the severity of AN. We suggest that these deficits and their impact on eating disorder symptoms should be taken into account in the treatment of individuals with AN. Individuals with AN could benefit from interventions such as cognitive remediation and emotion skills training that focus on both others’ and one’s own emotion identification and social cognition, or from mentalization-based treatment [37]. Difficulties in emotion recognition may cause struggles in friendships and other social relationships, and thus individuals with AN may also need extra support for engaging in these relationships.

## What is already known on this subject?

AN is associated with difficulties in emotion recognition, alexithymia, empathy, and emotion regulation. These difficulties may predispose to AN and contribute to the eating disorder symptoms and illness severity in individuals with AN. Comorbid conditions are common in individuals with AN, and it is unclear, whether challenges in emotion recognition, alexithymia, empathy, and emotion regulation reflect the combined effect of comorbid symptoms in individuals with AN.

## What does this study add?

We found that individuals with AN have challenges in complex emotion recognition and that these challenges occur independently of comorbid symptoms. In contrast, challenges in basic emotion recognition, alexithymia, empathy, and emotion regulation were not independent from common comorbid conditions in individuals with AN. We found that emotion recognition difficulties and high alexithymia may contribute to eating disorder behavior. Further, we found that increased emotion regulation difficulties predict a shorter duration of illness, higher body mass index, and increased eating disorder symptoms.

## Supplementary Information

Below is the link to the electronic supplementary material.

Supplementary file1 (DOCX 18 KB)

Supplementary file2 (DOCX 16 KB)

## Data Availability

The data of the study are available from the corresponding author upon reasonable request.
