# Supplementary material for: Emotion recognition, alexithymia, empathy, and emotion regulation in women with anorexia nervosa
Source: Eat Weight Disord. 2022 Oct 18;27(8):3587–97. doi: 10.1007/s40519-022-01496-2 (PMC9803740; doi:10.1007/s40519-022-01496-2)
Supplement: Supplementary file 1 — Supplementary file1 (DOCX 18 KB) [file 40519_2022_1496_MOESM1_ESM.docx]

|  | AN  (n = 42) | | | | | HC  (n = 40) | | | | |
| --- | --- | --- | --- | --- | --- | --- | --- | --- | --- | --- |
|  | **Minimum** | **Maximum** | **Mean** | **Median** | **Mode** | **Minimum** | **Maximum** | **Mean** | **Median** | **Mode** |
| Age (years) | 18.33 | 30.32 | 23.61 | 22.57 | 20.43 | 19.16 | 30.32 | 23.10 | 21.72 | 19.16 |
| Education (years) | 11 | 20 | 14.21 | 13.50 | 12 | 12 | 18 | 14.15 | 14.00 | 12 |
| FSIQ | 71 | 144 | 108.31 | 109.00 | 122 | 87 | 131 | 109.68 | 108.00 | 106 |
| PRI | 73 | 140 | 105.33 | 108.00 | 116 | 73 | 130 | 106.10 | 106.00 | 100 |
| VCI | 81 | 134 | 108.36 | 110.00 | 104 | 80 | 147 | 110.48 | 110.00 | 110 |
| BMI | 11.50 | 22.31 | 17.00 | 16.87 | 13.50 | 18.87 | 23.93 | 21.61 | 21.57 | 20.52 |
| Duration of illness | 2.33 | 16.42 | 7.46 | 6.80 | 3.58 | N/A | N/A | N/A | N/A | N/A |
| Eating Disorder symptoms total (EDE-Q) | 25 | 131 | 85.86 | 87.00 | 52 | 0 | 25 | 9.32 | 8.00 | 8 |
| Anxiety symptoms (BAI) | 3 | 45 | 19.31 | 17.00 | 14 | 0 | 12 | 4.83 | 4.00 | 4 |
| Depressive symptoms (BDI-II) | 1 | 51 | 23.52 | 22.50 | 14 | 0 | 15 | 2.45 | 1.50 | 0 |
| Obsessive-compulsive symptoms (OCI-R) | 7 | 55 | 22.93 | 20.50 | 16 | 1 | 20 | 7.43 | 5.50 | 2 |
| Autism spectrum traits (AQ) | 2 | 43 | 21.57 | 19.50 | 16 | 1 | 20 | 10.90 | 9.00 | 5 |

Supplementary Table 1. Descriptive statistic of background variables.

Abbreviations: FSIQ: Full-Scale Intelligence Quotient, VCI: Verbal Comprehension Index, PRI: Perceptual Reasoning Index, BMI: Body Mass Index, EDE-Q: Eating Disorder Examination Questionnaire, BAI: Beck Anxiety Inventory, BDI-II: Beck Depressive Inventory-II, OCI-R: Obsessive-Compulsive Inventory-Revised, AQ: Autism Quotient, N/A: Not Applicable, NS: Not Significant
