# Supplementary material for: Emotion recognition, alexithymia, empathy, and emotion regulation in women with anorexia nervosa
Source: Eat Weight Disord. 2022 Oct 18;27(8):3587–97. doi: 10.1007/s40519-022-01496-2 (PMC9803740; doi:10.1007/s40519-022-01496-2)
Supplement: Supplementary file 2 — Supplementary file2 (DOCX 16 KB) [file 40519_2022_1496_MOESM2_ESM.docx]

|  | AN  (n = 42) | | | | | HC  (n = 40) | | | | |
| --- | --- | --- | --- | --- | --- | --- | --- | --- | --- | --- |
|  | **Minimum** | **Maximum** | **Mean** | **Median** | **Mode** | **Minimum** | **Maximum** | **Mean** | **Median** | **Mode** |
| Basic emotion recognition from face pictures | 14 | 33 | 25.50 | 26.00 | 28 | 20 | 34 | 27.97 | 28.50 | 29 |
| Complex emotion recognition from face pictures | 28 | 59 | 47.22 | 47.50 | 42 | 38 | 62 | 52.28 | 53.00 | 55 |
| Complex emotion recognition from face videos | 24 | 41 | 32.69 | 33.00 | 35 | 31 | 45 | 37.63 | 37.00 | 36 |
| TAS-20 (Toronto Alexithymia Scale) | 33 | 78 | 55.93 | 56.50 | 44 | 28 | 54 | 36.95 | 37.00 | 39 |
| EQ (Empathy Quotient) | 20 | 76 | 48.88 | 48.00 | 42 | 27 | 75 | 54.93 | 57.00 | 57 |
| DERS (Difficulties in Emotion Regulation Scale) | 75 | 161 | 106.95 | 103.00 | 113 | 47 | 112 | 68.50 | 67.00 | 67 |

Supplementary Table 2. Descriptive statistics of the results of emotion recognition tasks, TAS-20, EQ, and DERS.
